# Deep Learning-Based Violin Bowing Action Recognition

**DOI:** 10.3390/s20205732

**Published:** 2020-10-09

**Authors:** Shih-Wei Sun, Bao-Yun Liu, Pao-Chi Chang

**Affiliations:** 1 Deptartment of New Media Art, Taipei National University of the Arts, Taipei 11201, Taiwan; 2 Computer Center, Taipei National University of the Arts, Taipei 11201, Taiwan; 3 Deptartment of Communication Engineering, National Central University, Taoyuan 32001, Taiwan; byliu@g.ncu.edu.tw (B.-Y.L.); pcchang@ce.ncu.edu.tw (P.-C.C.)

**Keywords:** deep learning applications, human perceptual cognition, depth camera, inertial sensor, action recognition, decision level fusion, violin bowing actions

## Abstract

We propose a violin bowing action recognition system that can accurately recognize distinct bowing actions in classical violin performance. This system can recognize bowing actions by analyzing signals from a depth camera and from inertial sensors that are worn by a violinist. The contribution of this study is threefold: (1) a dataset comprising violin bowing actions was constructed from data captured by a depth camera and multiple inertial sensors; (2) data augmentation was achieved for depth-frame data through rotation in three-dimensional world coordinates and for inertial sensing data through yaw, pitch, and roll angle transformations; and, (3) bowing action classifiers were trained using different modalities, to compensate for the strengths and weaknesses of each modality, based on deep learning methods with a decision-level fusion process. In experiments, large external motions and subtle local motions produced from violin bow manipulations were both accurately recognized by the proposed system (average accuracy > 80%).

## 1. Introduction

In a contemporary musical performance on stage, the actions and posture of the performer play critical roles in their storytelling. Action analysis and recognition technology can augment the artistic value of a performance. Specifically, such technology can recognize the actions of a stage performer and then provide visual feedback, audio effects, and haptic responses that can assist the performer to more precisely execute improved actions. For violin performers in particular, data from mounted cameras and wearable Internet of Things devices can be analyzed in order to achieve automated recognition of the players’ bowing actions. The recognized actions can be used to trigger interactive audio-visual effects that can be employed in contemporary musical performances on stage.

Camera sensors [1,2,3] are used to capture the external motion of a user, and the spatiotemporal data captured in the camera’s field of view can be analyzed, in order to achieve action recognition. Infrared (IR)-based depth cameras [4,5] are used to capture the three-dimensional (3D) depth information of a human subject, and the subject’s 3D body parts or skeletal structure can be used to recognize those actions with large visible motion, in order to reduce noise engendered by changes in lighting conditions. To recognize actions with subtle motions, data that are captured from wearable inertial sensors, such as accelerometers and gyro sensors [6,7], are analyzed.

**Figure 1:**
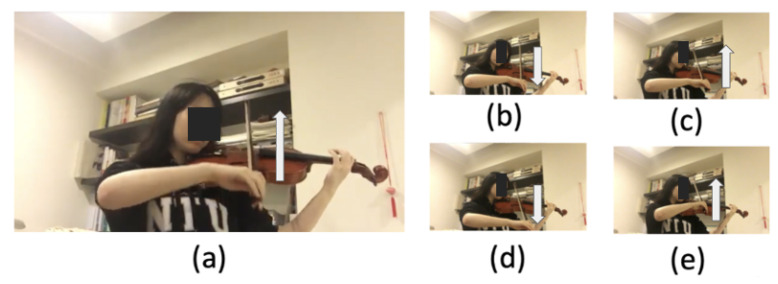
Violin bowing action and the movement of the right hand (white arrow): (**a**) a performer holding a violin to operate a bowing action, (**b**) first step of the “detache” bowing action, (**c**) second step of the detache bowing action, (**d**) third step of the detache bowing action, and (**e**) forth step of the detache bowing action.

In the recognition of violin bowing actions, large external motions and subtle local motions have equal value for interactive stage performances. However, research on action recognition has focused on large external motions but not on subtle local motions. Accordingly, this paper proposes a system that can recognize large and subtle violin bowing actions in order to fill this research gap. This system can be applied to interactive stage performances. In this system, bowing actions with large external motions (Figure 1) are captured from a camera, and bowing actions with subtle local motions are measured using wearable inertial sensors. Data on distinct motions of a performing violinist are collected and used to train action models. The trained models are then used for the automated recognition of the bowing actions.

The proposed system employs a fusion process to analyze data that are captured by the inertial sensors worn on a violin performer and those captured by the depth camera sensor. The contributions of this study are threefold: (1) a dataset comprising violin bowing actions was constructed from data captured by a depth camera and multiple inertial sensors; (2) data augmentation was achieved for depth-frame data through rotation in three-dimensional (3D) world coordinates and for inertial sensing data through yaw, pitch, and roll angle transformations; and, (3) bowing action classifiers were trained using different modalities, in order to compensate for the strengths and weaknesses of each modality, based on deep learning methods with a decision-level fusion process. The rest of this paper is organized, as follows: Section 2 discusses related work, Section 3 describes the bowing action dataset, Section 4 details the proposed bowing action recognition system, Section 5 presents the experimental results, and Section 6 concludes the paper.

## 2. Related Work

Research on action recognition has progressed from analyzing spatiotemporal information while using color cameras to analyze dynamic actions. For example, Zelnik-Manor and Irani [1] proposed a statistical distance measure for analyzing luminance data captured from a camera; the data were analyzed to cluster actions, such as “walk”, “Punch–Kick–Duck”, and “Tennis”. In addition, Khan and Sohn [2] proposed an approach that entails using a hidden Markov model to train models to analyze data from two color cameras. The trained models were used to recognize abnormal motions, which, in their study, were “forward fall”, “backward fall”, and “chest pain”. Additionally, Mehta et al. [3] proposed a system involving the use of a red–blue–green (RGB) camera to recognize human poses in 3D, such as those that are associated with “eating”, “sitting down”, and “taking a photo”.

Researchers have also used IR-based depth cameras (such as those in the Xbox Kinect) for human action recognition. Shotten et al. [4] proposed a method for analyzing depth frames captured from a Kinect camera with the body parts labeled; for such analysis, they used randomized decision trees and forests to train the classifiers in order to estimate human poses. To obtain greater detail from depth cameras, Dou et al. [5] utilized multiple RGBD cameras to estimate nonrigid motion fields for live performance capturing; their set of motions included “ taekwondo moves”, “dancing”, and “changing clothes”. However, the aforementioned actions involve large, easily recognizable body motions, especially as captured from a color or depth camera.

Camera-captured data are not suitable for the automated recognition of subtle motion, and data from wearable inertial sensors can be used instead. Several studies have adopted this approach. For example, Xie and Cao [6] formulated a method for hand gesture recognition, where accelerometer signals are analyzed by models trained using neural networks. Furthermore, Gupta et al. [7] used accelerometers and gyro sensors to detect the start and end points of meaningful gesture segments for hand gesture recognition. Chen et al. [8] also formulated a fusion process that was applied to both depth camera and inertial sensor signals for human action recognition; they analyzed actions, such as “clapping”, “throwing”, and “boxing”. In addition, Dawar et al. [9] proposed a deep learning–based fusion method; they used data from wearable inertial sensors and a depth camera to train models for recognizing actions, such as “smart TV interactions” and “transition movements” as well as human actions with large motions. In summary, sensor-based approaches [6,7] focus on recognizing subtle motion, such as hand gestures, and a combination of inertial sensors and a depth camera [8,9] can be used to recognize everyday actions that feature large motions.

When compared with everyday actions, violin bowing is characterized by subtle motions. Dalmazzo and Ramirez [10] formulated a method for recognizing such subtle motions; in this method, electromyography (EMG) signals from the violinist’s forearm are analyzed for finger gesture recognition. Another study classified bowing gestures by applying machine learning [11] to data from inertial sensors and audio recordings. The shortcoming of the first study lies in the unreliability of EMG; EMG signals [10] vary depending on which part of the forearm the sensor is located. The shortcoming of the second study lies in its small dataset: the data were gathered from one performer who repeatedly executed only a limited variety of bowing motions [11].

Most everyday actions—such as the “taekwondo moves”, “dancing”, and “clapping” analyzed in previous studies—feature large motions of body parts, such as the hands, feet, and upper body. By contrast, a performing violinist adopts either a sitting or standing posture and manipulates the bow using subtle hand movements. Thus, the automated recognition of violin bowing actions is challenging because of such subtlety in motion and the large motion properties in bowing action, which makes datasets on violin bowing actions difficult to construct. To meet this challenge, we propose a system entailing the use of a depth camera to capture large motions and the use of a wearable sensors to capture subtle motions. We captured data from a violinist playing complete pieces. Our proposed violin bowing action recognition system can be used for musical performances that involve the violin.

## 3. Bowing Action Dataset Collected from a Violinist Performing Complete Pieces

According to Dalmazzo and Ramirez [11], violin performance typically involves the bowing techniques of “detache”, “legato”, “sautille”, and “spiccato”. In addition, the up bow and down bow are the basic bowing actions that violinists use to trigger audio–visual effects in a stage performance. Therefore, we focused on recognizing the six basic, representative violin bowing actions: detache, legato, sautille, spiccato, up bow, and down bow. A typical violin performer holds the violin and bow using their left and right hands, respectively, as depicted in Figure 1a. For example, the detache bowing action, as depicted from Figure 1b–e, is executed in sequence. Most studies on violin bowing action recognition, such as [11], have only focused on violin bowing. However, performers in our study were invited to play complete pieces. Subsequently, a segmentation process was conducted by an expert in violin performance. In other words, the violin bowing action dataset was generated from the segments of the performances of complete pieces by human violinists.

**Figure 2:**
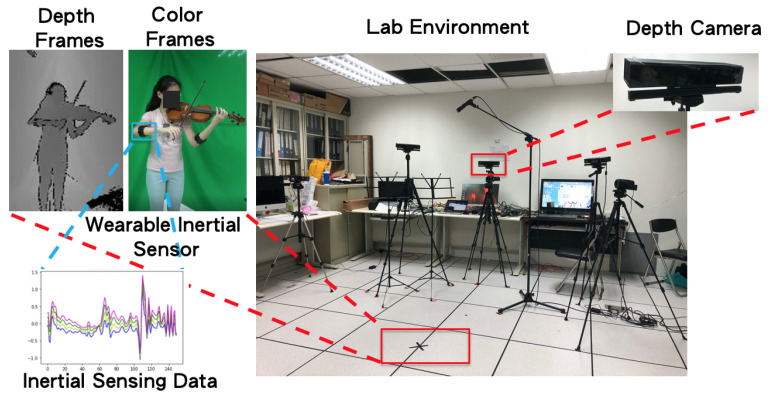
Lab environment for recording violin bowing actions.

**Figure 3:**
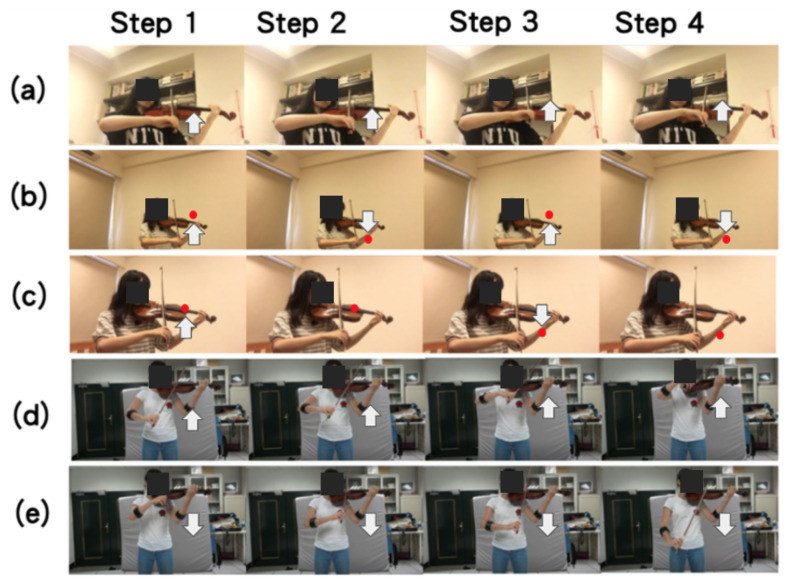
Examples of violin bowing actions: (**a**) legato, (**b**) sautille, (**c**) spiccato, (**d**) up bow, and (**e**) down bow. White arrows represent the movement of the hand when manipulating the bow, and red dots represent the player temporally dragging and leaving the violin in a short period.

In the proposed violin bowing action dataset, we focus on subtle motions and large motions, as executed by performing violinists. As illustrated in Figure 2, one depth camera (Kinect V2, Microsoft, Redmon, WA, USA [12]) was used to capture depth frames and color frames (Figure 2, left panel). In addition, a wearable inertial sensor (a Myo sensor [13], as indicated by the blue rectangle in the middle panel of Figure 2), was worn on the performer’s right hand. The performer stood at a position that was marked by a black cross (Figure 2, bottom red rectangle in right panel) to play complete violin pieces. Inertial sensing data were recorded when the performer’s right hand manipulated the bow (Figure 2, bottom left panel). The data in our bowing action dataset were captured in sync by only one depth camera (Figure 2, upper red rectangle in right panel) and one wearable inertial sensor (Figure 2, blue rectangle in central panel). Figure 3a–e depict the legato, sautille, spiccato, up bow, and down bow bowing actions.

The data were captured from eight violin performers, who majored in violin from the Department of Music of Taipei National University of the Arts (TNUA), Taiwan. The participating violinists were invited to play ten distinct and complete violin pieces. Subsequently, an expert in violin performance was invited to label the bowing actions at the corresponding time tags for video segmentation. Finally, segments with depth frames and color frames and the corresponding inertial sensing data (i.e., signals from the gyro sensor and accelerometer) could be obtained. We collected data on the six types of bowing actions, and each action had six segments, each of which was played by each of the eight violin performers.

## 4. Proposed Deep Learning–Based Violin Bowing Action Recognition System

**Figure 4:**
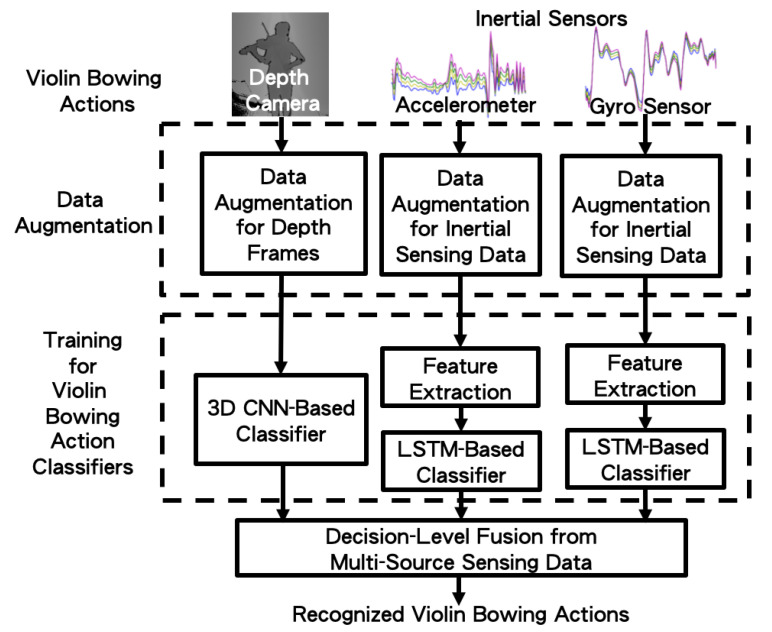
The system block diagram of the proposed method.

The single depth camera and two inertial sensors used to capture the data are depicted in the top panels of Figure 4. Before the classifiers could be trained to identify violin bowing actions, due to the limited action samples in our collected dataset, we must perform data augmentation in order to enlarge the number of training samples from the collected sensing data. Subsequently, deep learning methods were used to train models to identify each performer’s violin bowing actions. Finally, we applied our proposed probabilistic decision fusion process to the multi-source sensing data in order to achieve the automated recognition of bowing actions. Our proposed method is detailed, as follows.

### 4.1. Data Augmentation

We conducted data augmentation to increase the variety of the samples of violin bowing actions in the limited dataset. The data augmentation method was motivated by that of Dawar et al. [9].

#### 4.1.1. Data Augmentation for Depth Frames

The pixel values and positions of a depth frame represent information on the 3D world coordinates of the captured body part of a violin player. To augment the limited number of recorded depth frames, the camera’s position can be rotated in 3D in order to obtain more violin performance samples. As defined in [14], given a pixel depth value *Z* at a specific pixel (x,y) in a depth frame of focal lengths $f_{x}$ and $f_{y}$, and center position $\left(C_{x},C_{y}\right)$, the 3D world coordinates (X,Y,Z) can be obtained while using the following equation [14]:

(1) $$\begin{array}{c} X=Z\left(x-C_{x}\right)/f_{x},\\ Y=Z\left(y-C_{y}\right)/f_{y}. \end{array}$$

As suggested by [9], for the Kinect v2 depth camera used in this study, the values of $f_{x}$ and $f_{y}$ were set to 365 and 365, respectively.

In addition, the rotations of the camera about the *x*, *y*, and *z* axes, as denoted α, β, and γ, can be represented according to the transformation matrices defined in [14], written as follows:

(2) $$R_{T_{x}}=\left[\begin{array}{cccc} 1 & 0 & 0 & 0\\ 0 & cos(\alpha ) & -sin(\alpha ) & Z\cdot sin(\alpha )\\ 0 & sin(\alpha ) & cos(\alpha ) & Z\cdot (1-cos\alpha )\\ 0 & 0 & 0 & 1 \end{array}\right],$$

(3) $$R_{T_{y}}=\left[\begin{array}{cccc} cos(\beta ) & 0 & sin(\beta ) & -Z\cdot (sin(\beta ))\\ 0 & 1 & 0 & 0\\ -sin(\beta ) & 0 & cos(\beta ) & Z\cdot (1-cos\beta )\\ 0 & 0 & 0 & 1 \end{array}\right],$$

(4) $$R_{T_{z}}=\left[\begin{array}{cccc} cos(\gamma ) & -sin(\gamma ) & 0 & 0\\ sin(\gamma ) & cos(\gamma ) & 0 & 0\\ 0 & 0 & 1 & 0\\ 0 & 0 & 0 & 1 \end{array}\right].$$

The new coordinates $\left(X^{\prime },Y^{\prime },Z^{\prime }\right)$ after the rotations are as follows:

(5) $$\left[\begin{array}{c} X^{\prime }\\ Y^{\prime }\\ Z^{\prime }\\ 1 \end{array}\right]=R_{T_{x}}R_{T_{y}}R_{T_{z}}\left[\begin{array}{c} X\\ Y\\ Z\\ 1 \end{array}\right].$$

Data augmentation for the depth frames can be obtained according to the given values of α, β, and γ. The new pixels can be derived when the transformed 3D world coordinates are obtained:

(6) $$\begin{array}{c} x^{\prime }=\left(\left(X^{\prime }\cdot f_{x}\right)/Z^{\prime }\right)+C_{x},\\ y^{\prime }=\left(\left(Y^{\prime }\cdot f_{y}\right)/Z^{\prime }\right)+C_{y}. \end{array}$$

**Figure 5:**
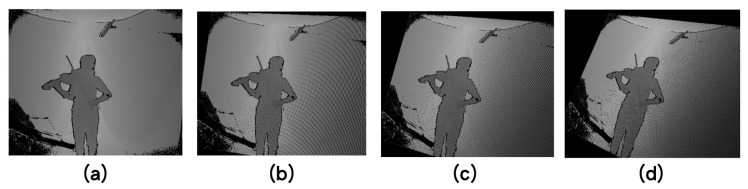
Data augmentation for depth frames: (**a**) original depth frame, (**b**) $\left(\alpha ,\beta ,\gamma \right)=\left(5^{\circ },5^{\circ },5^{\circ }\right)$, (**c**) $\left(\alpha ,\beta ,\gamma \right)=\left(10^{\circ },10^{\circ },10^{\circ }\right)$, and (**d**) $\left(\alpha ,\beta ,\gamma \right)=\left(15^{\circ },15^{\circ },15^{\circ }\right)$.

Examples of data augmentation for the depth frames are presented in Figure 5.

#### 4.1.2. Data Augmentation for Inertial Sensing Data

The sensor-recorded data represent the yaw, pitch, and roll angles of the inertial sensor worn on the violinist’s hand while manipulating the bow. We can assign additional 3D rotation angles to obtain more samples in the inertial sensor modality to augment the recorded sensing data. For the inertial sensing data captured by the accelerometer, let $\theta_{a}$, $\alpha_{a}$, and $\varphi_{a}$ represent the yaw, pitch, and roll variations about the three axes, respectively. According to the definition in [15], the rotation transformation matrices are as follows:

(7) $$R_{a_{x}}=\left[\begin{array}{ccc} 1 & 0 & 0\\ 0 & cos(\theta_{a}) & -sin(\theta_{a})\\ 0 & sin(\theta_{a}) & cos(\theta_{a}) \end{array}\right],$$

(8) $$R_{a_{y}}=\left[\begin{array}{ccc} cos(\varphi_{a}) & 0 & sin(\varphi_{a})\\ 0 & 1 & 0\\ -sin(\varphi_{a}) & 1 & cos(\varphi_{a}) \end{array}\right],$$

(9) $$R_{a_{z}}=\left[\begin{array}{ccc} cos(\rho_{a}) & -sin(\rho_{a}) & 0\\ sin(\rho_{a}) & cos(\rho_{a}) & 0\\ 0 & 0 & 1 \end{array}\right].$$

Given the original three-axis accelerometer sensing values $a_{x}$, $a_{y}$, and $a_{z}$, the new acceleration values $a_{x}^{\prime }$, $a_{y}^{\prime }$, and $a_{z}^{\prime }$ can be obtained, as follows:

(10) $$\left[\begin{array}{c} a_{x}^{\prime }\\ a_{y}^{\prime }\\ a_{z}^{\prime } \end{array}\right]=R_{a_{x}}R_{a_{y}}R_{a_{z}}\left[\begin{array}{c} a_{x}\\ a_{y}\\ a_{z} \end{array}\right].$$

Similarly, according to the definition of Yurtman and Barshan [15], the transform rotation matrix for the gyro sensor signals is as follows:

(11) $$R_{g_{x}}=\left[\begin{array}{ccc} 1 & 0 & 0\\ 0 & cos(\theta_{g}) & -sin(\theta_{g})\\ 0 & sin(\theta_{g}) & cos(\theta_{g}) \end{array}\right],$$

(12) $$R_{g_{y}}=\left[\begin{array}{ccc} cos(\varphi_{g}) & 0 & sin(\varphi_{g})\\ 0 & 1 & 0\\ -sin(\varphi_{g}) & 1 & cos(\varphi_{g}) \end{array}\right],$$

(13) $$R_{g_{z}}=\left[\begin{array}{ccc} cos(\rho_{g}) & -sin(\rho_{g}) & 0\\ sin(\rho_{g}) & cos(\rho_{g}) & 0\\ 0 & 0 & 1 \end{array}\right].$$

Given the original gyro sensor values $g_{x}$, $g_{y}$, and $g_{z}$ for the three axes, the new gyro sensor values $g_{x}^{\prime }$, $g_{y}^{\prime }$, and $g_{z}^{\prime }$ can be obtained, as follows:

(14) $$\left[\begin{array}{c} g_{x}^{\prime }\\ g_{y}^{\prime }\\ g_{z}^{\prime } \end{array}\right]=R_{g_{x}}R_{g_{y}}R_{g_{z}}\left[\begin{array}{c} g_{x}\\ g_{y}\\ g_{z} \end{array}\right].$$

**Figure 6:**
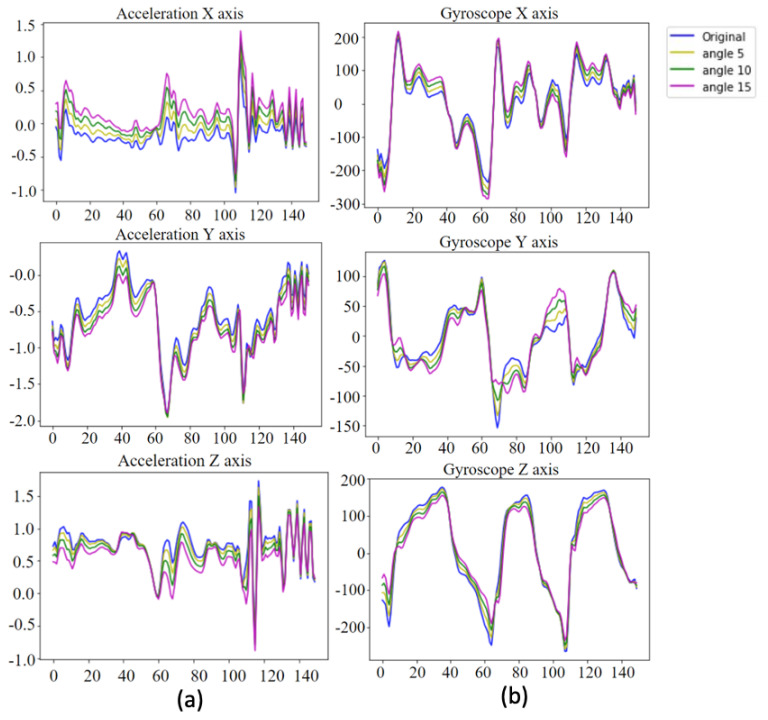
Data augmentation for inertial sensing data: (**a**) accelerometer and (**b**) gyro sensor.

In Figure 6a, the blue curves represent the original inertial sensing data for the accelerometer, and the yellow, green, and pink curves represent examples of the data augmentation results for the assigned rotation values of $\left(\theta_{a},\varphi_{a},\rho_{a}\right)$, where $\left(5^{\circ },5^{\circ },5^{\circ }\right)$, $\left(10^{\circ },10^{\circ },10^{\circ }\right)$, and $\left(15^{\circ },15^{\circ },15^{\circ }\right)$. Similarly, Figure 6b shows the data augmentation results for the gyro sensor subjected to violin bowing actions.

### 4.2. Training of Violin Bowing Action Classifier

Using the augmented data from the depth frames and inertial sensors, we separately trained violin bowing action classifiers for the various modalities, as shown by the three vertical paths that were bounded by the bottom dashed blocks in Figure 4. For the depth camera modality, the classifiers were trained using the 3D convolutional neural network (3D-CNN) [16]. For the inertial sensor modality, the classifiers were trained using long short-term memory (LSTM) networks [17].

#### 4.2.1. 3D-CNN-Based Classifiers for Depth Frames

**Figure 7:**
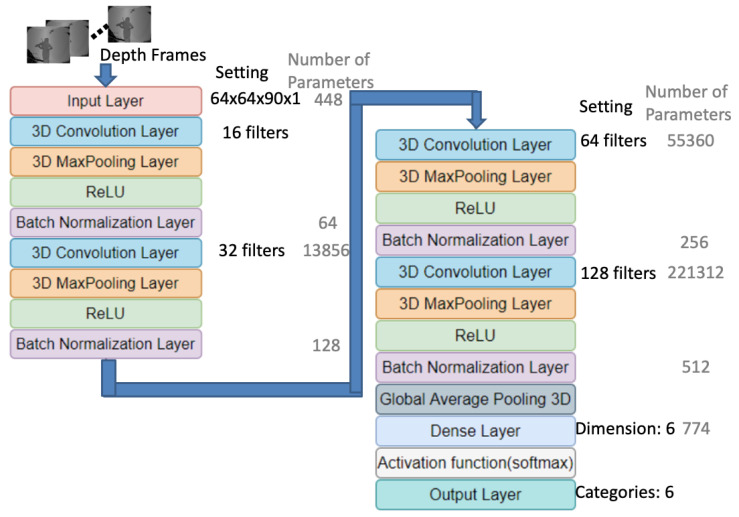
Three-dimensional convolutional neural network (3D-CNN)-based training process for depth frames [16] with global average pooling [18] (blue–dark gray block) and batch normalization [19] (purple blocks).

The depth frames that were augmented with new pixels through Equation (6), as displayed in the top left frames of Figure 7, were resized to a resolution of 64×64 before being fed into the adopted 3D-CNN model [16]. The frame rate of the depth camera was 30 frames per second (FPS), and the manually labeled violin bowing actions (by experts in violin performance) constituted less than 1.5 s (at 90 frames) of the data. Thus, for each violin bowing segment, the number of frames were resampled to 90. Moreover, only one gray-level image channel was used for representing the depth frames. Therefore, a 64×64×90×1 tensor was treated as the input of the 3D-CNN model (Figure 7, pink block in the left panel). The filter settings of the 3D-CNN layers are also listed in Figure 7: stride = 1; padding = “same”; and, convolutional kernel size = 3×3×3. Furthermore, an Adam optimizer and categorical crossentropy loss function were used to implement the 3D-CNN model. Because we attempted to recognize six violin bowing actions, the dimension of the dense layer (light blue block) of the 3D-CNN architecture was set to 6 (Figure 7, bottom right panel). In addition, we added a global average pooling [18] layer at the end of the adopted 3D-CNN model in order to prevent overfitting (Figure 7, blue–dark gray block in bottom right panel). After the dense layer, a softmax activation function (light gray block) was used to output the classification results, which ranged from 0 to 1. Moreover, we added batch normalization layers [19] at the end of each 3D convolution layer in order to reduce overfitting (Figure 7, purple blocks).

#### 4.2.2. LSTM-Based Classifiers for Inertial Sensing Data

**Figure 8:**
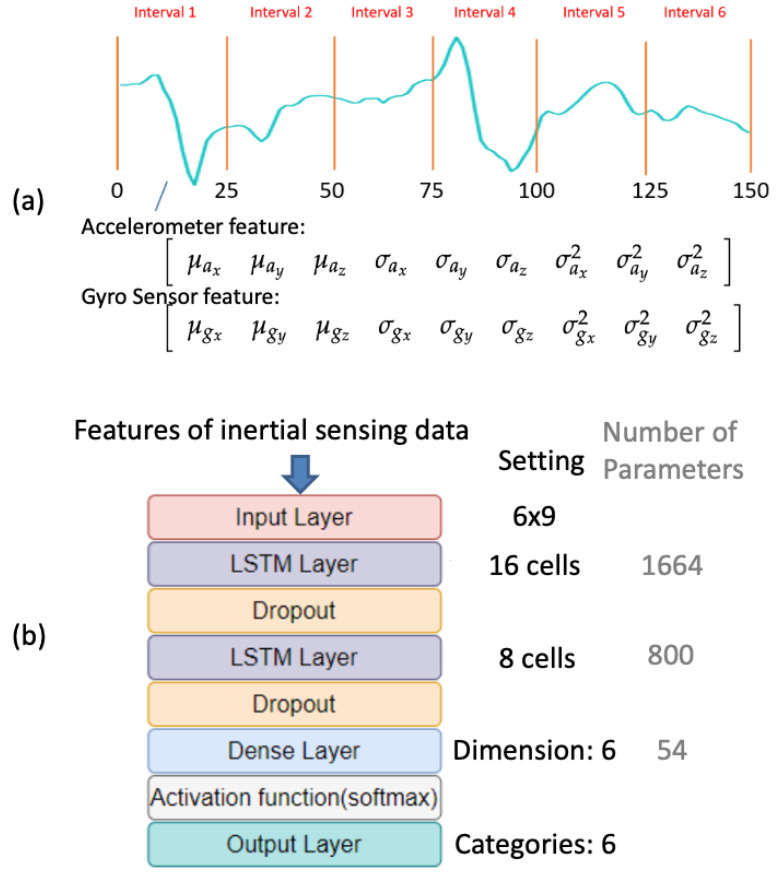
Training processes for the inertial sensing data: (**a**) feature extraction; (**b**) training process of the adopted long short-term memory (LSTM) [17].

The augmented inertial sensing data that were obtained from the accelerometer and gyro sensor modalities were resampled to 150 samples, and their sequences were segmented to six intervals [8]. On the basis of the procedures in our previous work [20], we calculated the statistical features of the modalities in each interval (Figure 8a). For example, in Interval 1 (Figure 8a), the statistical features of the accelerometer modality in the x-axis were represented by the mean $\mu_{a_{x}}$, standard deviation $\sigma_{a_{x}}$, and variance $\sigma_{a_{x}}^{2}$. Therefore, the features of the accelerometer modality in the x-, y-, and z-axes in the first interval could be evaluated, as follows (Figure 8a, bottom left panel):

(15) $$\alpha_{t=1}=\left[\begin{array}{ccccccccc} \mu_{a_{x}} & \mu_{a_{y}} & \mu_{a_{z}} & \sigma_{a_{x}} & \sigma_{a_{y}} & \sigma_{a_{z}} & \sigma_{a_{x}}^{2} & \sigma_{a_{y}}^{2} & \sigma_{a_{z}}^{2} \end{array}\right].$$

Subsequently, the features could be calculated at t=2,t=3,⋯,t=6 and concatenated as a 6×9 tensor, which was used as the input of the LSTM [17] model (Figure 8b, top panel). The dimension of the dense layer (Figure 8b, light blue block) of the LSTM model was set to 6 in order to generate the violin bowing action classifiers for the six actions (Figure 8b, bottom panel). The softmax activation function (Figure 8b, light gray block) was used after the dense layer to constrain the classification output in the range from 0 to 1. In addition, the hidden units of the proposed method were set to 16 and 8 (Figure 8b, black font) for the first and second LSTM layers, respectively. Similarly, the statistical features for the gyro sensor modality in the x-, y-, and z-axes at t=1 were

(16) $$\eta_{t=1}=\left[\begin{array}{ccccccccc} \mu_{g_{x}} & \mu_{g_{y}} & \mu_{g_{z}} & \sigma_{g_{x}} & \sigma_{g_{y}} & \sigma_{g_{z}} & \sigma_{g_{x}}^{2} & \sigma_{g_{y}}^{2} & \sigma_{g_{z}}^{2} \end{array}\right],$$

and the same LSTM neural network architectures shown in Figure 8b were used to train the classifiers for the gyro sensor modality. Figure 7 and Figure 8b (gray font) show the resulting number of parameters trained for the 3D-CNN and LSTM architectures, respectively.

### 4.3. Decision-Level Fusion from Multi-Source Sensing Data

For the classifier trained using the depth frame modality and those trained using the accelerometer and gyro sensor modalities, output scores were produced, which ranged from 0 to 1. These scores represented the output probabilities for each class of violin bowing actions. On the basis of the linear opinion pool that was proposed by Li et al. [21] for decision fusion, we adopted conditional class probabilities, as defined in [22], in our method in order to estimate a weighted product of these output probabilities for multiple sensors. The final class label τ could be derived while using the following equation:

(17) $$\begin{array}{c} \tau ={\arg\max}_{i=1,\cdots ,C}P\left(\tau_{k}|\kappa \right), \end{array}$$

where *C* is the number of classes and *i* is the index of the violin bowing actions (classes); the global membership function is as follows:

(18) $$\begin{array}{c} P\left(\tau_{i}|\kappa \right)=\prod_{q=1}^{Q}p_{q}{\left(\tau_{i}|\kappa \right)}^{\alpha_{q}}, \end{array}$$

where ${\left\{\alpha_{q}\right\}}_{q=1}^{Q}$ represents the classifier weights uniformly distributed over all classifiers. In Equation (18), *Q* is the number of classifiers and $p_{q}\left(\tau_{i}|\kappa \right)$ is the output probability for the class $\tau_{k}$ according to input κ. In this paper, the parameter *Q* was set to three for the three sensor modalities (depth camera, accelerometer, and gyro sensor); moreover, $\alpha_{q}=\frac{1}{Q}=\frac{1}{3}$ was used for the uniformly distributed weights.

## 5. Experimental Results

**Figure 9:**
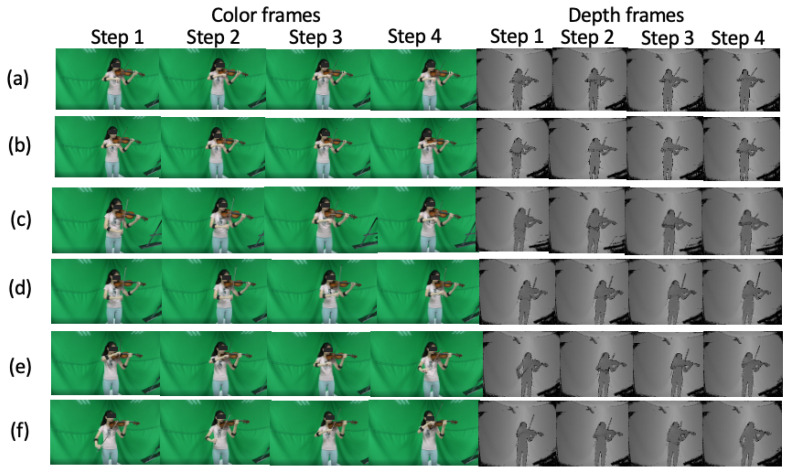
Color frames and depth frames collected from the Kinect V2 [12] camera: (**a**) datache, (**b**) legato, (**c**) sautille, (**d**) spiccato, (**e**) up bow, and (**f**) down bow.

**Figure 10:**
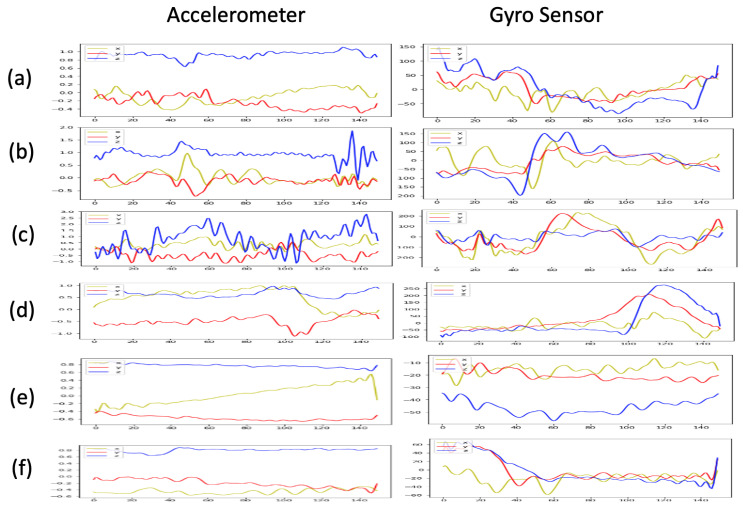
Inertial sensing data collected from the Myo sensor [13]: (**a**) datache (**b**) legato, (**c**) sautille, (**d**) spiccato, (**e**) up bow, and (**f**) down bow.

Experiments were conducted in order to validate the effectiveness of our proposed deep learning-based violin bowing action recognition system. We constructed a violin bowing action dataset by gathering data using a Kinect V2 [12] depth camera and Myo sensor [13] worn by a performer (Figure 2). Figure 9 shows the corresponding color frames and depth frames for the six violin bowing actions. The camera-captured depth frames (the right part of Figure 9) were used to train the bowing action classifiers. Because the unstable lighting conditions in a stage performance may introduce noise in the obtained color frames, the color frames in our experiment (the left part of Figure 9) should only be used as a reference. In addition, Figure 10 presents the inertial sensing data provided by the accelerometer and gyro sensor for the six bowing actions. Moreover, the Kinect camera recorded the depth frames at 30 FPS, and the Myo sensor recorded the inertial sensing data (from the accelerometer and gyro sensor) at 50 Hz. Eight violin performers were invited to play 10 complete pieces to collect the bowing action dataset: Bach’s Partita No. 2 for Solo Violin, Mozart’s Violin Concerto No. 3, and Beethoven’s Violin Sonata No. 5. Subsequently, one of the eight violin performers was invited to extract the bowing segments and label the bowing actions as the ground truth. For each bowing action, six segments were extracted with the depth frames, accelerometer data, and gyro sensor data for each performer. Therefore, a dataset with 8 (violin performers) × 6 (violin bowing actions) × 6 (segments) = 288 violin bowing action samples was generated and used in the experiments. In the experiments, we adopted leave-one-out cross-validation [23]. We used the data of one, one, and six of the eight violin performers for testing, validation, and training, respectively.

### 5.1. Quantitative Evaluation

**Figure 11:**
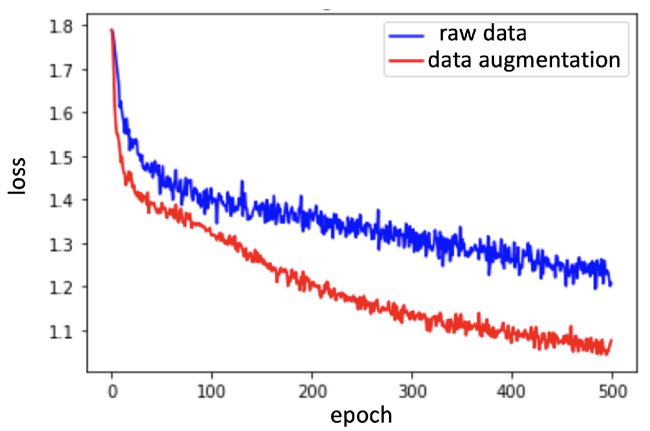
Loss measured from the obtained models with the raw data of both the depth frames and inertial sensing data (blue curve) and with data augmentation (red curve).

**Figure 12:**
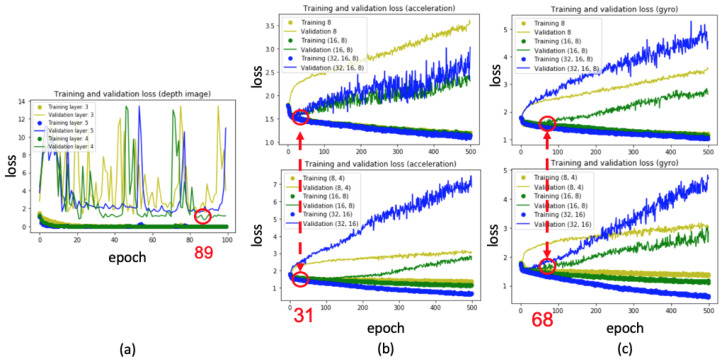
Training and validation losses for Violin Performer 8: (**a**) 3D-CNN training for depth-camera data (using the trained model at epoch 89), (**b**) LSTM training for accelerometer data (using the trained model at epoch 31), and (**c**) LSTM training for gyro sensor data (using the trained model at epoch 68).

In the experiments, based on the Tensorflow [24] and Keras [25] libraries, the 3D-CNN [16] and LSTM [17] models were implemented to train the classifiers to recognize violin bowing actions. To train the 3D-CNN classifier using depth frames, four hidden layers and a learning rate of 0.001 were employed; training was evaluated over 100 epochs. To train the LSTM classifier while using the inertial sensor data, two hidden layers (16 cells and eight cells), a learning rate of 0.001, and a dropout rate of 50% were employed; training was evaluated over 500 epochs. Moreover, the models with the lowest validation loss were saved as the final model. According to the data augmentation process that is described in Section 4.1, we used the three parameters of $\left(5^{\circ },5^{\circ },5^{\circ }\right)$, $\left(10^{\circ },10^{\circ },10^{\circ }\right)$, and $\left(15^{\circ },15^{\circ },15^{\circ }\right)$ to generate an augmented sensing dataset. The original dataset had 288 samples and the augmented dataset had 288×3=864 samples, as described in Section 5. As indicated by the red curve in Figure 11, the overall training loss was reduced relative to the loss when only raw data were used (blue curve in Figure 11). Furthermore, the training and validation losses for the augmented data are shown in Figure 12a–c. In Figure 12a, 3D-CNN layers are trained from three layers to five layers, and the green curve with four 3D convolution layers achieves the lowest validation loss. Therefore, four 3D convolution layers are used, as shown in Figure 7. Similarly, in Figure 12b,c, LSTM layers are trained from one layer to three layers with different cells, and two LSTM layers with (16,8) cells (green curve) achieves the lowest validation loss. Finally, two LSTM layers with (16,8) cells are used, as shown in Figure 8b. In addition, the model with the lowest validation loss was taken as the final model for bowing action classification in order to avoid the overfitting issues, as marked by the red circles in Figure 12a–c.

Table 1 lists the accuracy rates of the leave-one-out cross-validation [23] process for the eight violin performers. and the average accuracy rates are listed in the final row of this table. Sensing data that were obtained from one of the violin performers were selected for testing, data from another one performer for validation, and those that were obtained from the other performers were used for training. The accuracy rates of the classifiers trained using the 3D-CNN model and the depth frames [16] (described in Section 4.2.1) are shown in the second column of Table 1; the accuracy rates of the classifiers trained using the LSTM model [17] (as described in Section 4.2.2) and the accelerometer and gyro sensor data are listed in the third and fourth columns of Table 1, respectively. As described in Section 4.3, the decision-level fusion method was used, and the fusion results for the two modalities are presented in the fifth to seventh columns of Table 1. Finally, the fusion results for all three modalities are presented in the last column of Table 1. In general, the results that were obtained using the three modalities (depth frames + accelerometer + gyro sensor) had the highest average accuracy (Table 1, bottom right part). However, for Violin Performer 4 and Violin Performer 5, the highest accuracy was achieved when two modalities (depth frames + accelerometer) were used; this was because the gyro sensor modality provided relatively low accuracy (at 25.0% and 19.4% for Violin Performers 4 and 5, respectively, relative to the D and A modalities). Similarly, for Violin Performer 6, the highest average accuracy was achieved when the depth frame and gyro sensor modalities were used; this was because the accelerometer modality had relatively low accuracy (27.8%).

**Figure 13:**
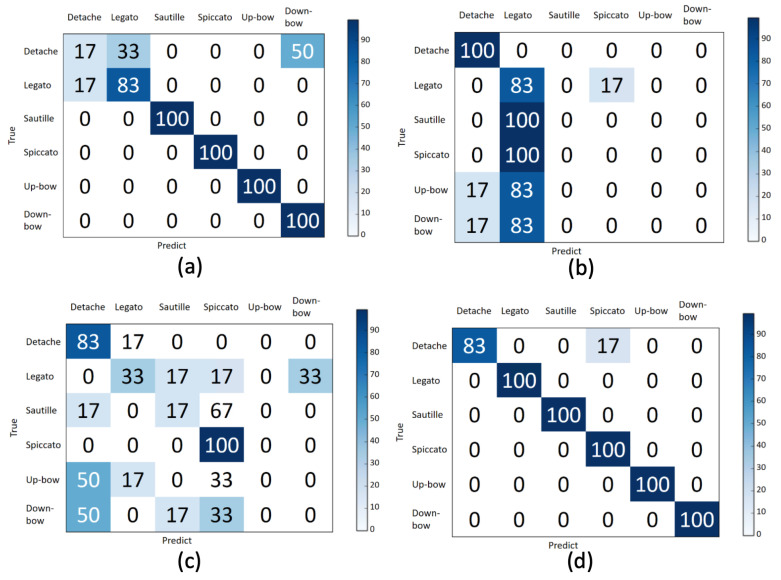
Confusion matrices for Violin Performer 8 for six bowing actions analyzed using the following modalities after applying decision-level fusion: (**a**) depth frames (D), (**b**) accelerometer (A), (**c**) gyro sensor (G), and (**d**) D + A + G.

Consider, for example, Violin Performer 8; the confusion matrices for the six bowing actions are illustrated in Figure 13a–d. In Figure 13, the actual results for the violin bowing actions are presented in the rows, and the predicted results are presented in the columns. The classifier trained by the 3D-CNN [16] model and the depth frames recognized most of the bowing actions, but tended to misclassify detache as legato and down bow in the depth frames, as shown in Figure 13a. However, although the accelerometer and gyro sensor modalities had noisy results for other bowing actions when the LSTM-based [17] classifier was used, the accelerometer and gyro sensor modalities resulted in an improved recognition of detache, as shown in Figure 13b,c. Nevertheless, the inertial sensor modality did not enhance the recognition performance for most bowing actions. However, for detache, the inertial sensor modality could compensate for the shortcomings of the depth camera modality. In general, choosing among the modalities to achieve optimal recognition performance was difficult. Nonetheless, upon applying the proposed decision-level fusion process that is described in Section 4.3, we noted that the recognition accuracy could be improved, as presented in Figure 13d.

### 5.2. Complexity Results

We executed our machine learning experiments in an Ubuntu 18.04 environment, with an Intel Core i7 CPU clocked at 3.60 GHz, an Nvidia RTX 2080 graphics card, and 64 GB of RAM. Table 2 presents the total computational time for the quantitative evaluations (Section 5.1). The process of collecting the 8 (violin performers) × 6 (violin bowing actions) × 6 (segments) =288 bowing action samples was temporally synchronized. The execution time for the leave-one-out cross-validation [23] is presented in Table 2. Because the use of 3D-CNN models for training requires the analysis of the spatiotemporal information for a segment of sequential depth frames, the computational time for the 3D-CNN model (Table 2, second column) was greater than those for the LSTM-based model used for the other two (accelerometer and gyro sensor) modalities (Table 2, third and fourth columns). Moreover, once the bowing action classifiers were established, the proposed decision-level fusion process was efficient, requiring approximately 5 s to test all of the samples (Table 2, last column). The inference time per sample is listed in the last column of Table 2; for a given bowing action sample, the recognition result was rapidly obtained (average execution time = 0.1444 s), rendering our system suitable for real-time applications.

## 6. Conclusions

Our proposed system can recognize violin bowing actions that feature large external motions and subtle local motions. In our system, 3D-CNN-based classifiers are applied to depth frames, and LSTM-based classifiers are applied to inertial sensor data. Most studies on bowing gesture recognition have not analyzed both large external motions and subtle local motions, which is essential to bowing action recognition; such studies have primarily focused on inertial sensing data and audio tracks. We analyzed six representative violin bowing actions, and our system, employing the decision-level fusion process, recognized the actions at a high average accuracy of 80%. Our contribution is threefold: (1) a dataset comprising violin bowing actions was generated from sensing data that were collected from a violinist performing complete pieces; (2) data augmentation was used to effectively generate action models from the limited sensing data; and, (3) a decision-level fusion method for 3D-CNN and LSTM models was proposed to train violin bowing action classifiers. Our preliminary results indicate that the use of a depth camera to capture large external motions and the use of inertial sensors to capture subtle local motions have their respective strengths and weaknesses; the joint use of both can allow each approach to compensate for the other’s weaknesses to enhance action recognition capabilities. In future work, measurements of the internal muscle movements of violinists can also be taken to capture bowing actions, and the resulting big sensing dataset can be analyzed using deep learning. For practitioners, our violin bowing action recognition system can be incorporated into interactive aural or visual artworks or into interactive stage performances.

## Figures and Tables

**Table 1:** Accuracy of different modalities.

	Depth Frames (D)	Accelerometer (A)	Gyro Sensor (G)	D + A	D + G	A + G	D + A + G
violin performer 1	55.6%	13.9%	30.6%	58.3%	66.6%	19.5%	61.1%
violin performer 2	91.7%	36.1%	36.1%	94.4%	94.4%	36.1%	97.2%
violin performer 3	66.7%	27.8%	30.6%	75.0%	66.7%	33.3%	80.6%
violin performer 4	97.2%	33.3%	25.0%	97.2%	91.6%	30.6%	94.4%
violin performer 5	66.7%	25.0%	19.4%	66.7%	63.9%	19.4%	63.9%
violin performer 6	75.0%	27.8%	33.3%	75.0%	86.1%	33.3%	68.1%
violin performer 7	61.1%	30.6%	33.3%	66.7%	58.3%	33.3%	66.7%
violin performer 8	83.3%	30.6%	38.9%	80.6%	86.1%	44.4%	97.2%
average	74.7%	28.1%	30.9%	76.7%	76.7%	31.3%	80.9%

**Table 2:** Computational time for data from depth frames (D), accelerometer (A), and gyro sensor (G).

Time (seconds)	Training for D	Training for A	Training for G	Testing for D+A+G	Inference Time per Sample
violin performer 1	607.7	444.3	455.1	4.3	0.1194
violin performer 2	607.0	445.1	455.2	4.3	0.1194
violin performer 3	638.9	448.1	460.0	4.6	0.1278
violin performer 4	611.1	448.3	467.0	5.0	0.1389
violin performer 5	610.2	451.1	462.0	5.3	0.1472
violin performer 6	613.0	450.9	461.7	5.7	0.1583
violin performer 7	671.2	452.5	462.1	6.1	0.1694
violin performer 8	615.7	453.7	465.0	6.5	0.1806
average	622.0	449.3	461.0	5.2	0.1444
